# Frailty, nutritional status, and inflammation as determinants of chemotherapy delivery and outcomes in pancreatic cancer patients receiving gemcitabine plus nab-paclitaxel

**DOI:** 10.3389/fonc.2026.1730394

**Published:** 2026-03-16

**Authors:** Ho Seung Lee, Chan Min Jeong, Jae Min Lee, Tae In Kim, Sang Hyun Kim, Han Jo Jeon, Hyuk Soon Choi, Eun Sun Kim, Bora Keum, Yoon Tae Jeen

**Affiliations:** 1 Department of Internal Medicine, Korea University Anam Hospital, Seoul, Republic of Korea; 2 College of Medicine, Korea University, Seoul, Republic of Korea

**Keywords:** chemotherapy dose intensity, frailty, neutrophil-to-lymphocyte ratio, pancreatic neoplasms, prognosis

## Abstract

**Introduction:**

Pancreatic cancer has high mortality, and optimizing chemotherapy delivery in frail patients is challenging. Frailty and systemic inflammation are increasingly recognized as prognostic factors; however, their roles in patients receiving gemcitabine plus nab-paclitaxel (GnP) are not well defined. We aimed to evaluate the impact of a composite frailty index (modified frailty index [mFI] ≥2 and prognostic nutritional index [PNI]<45) and neutrophil-to-lymphocyte ratio (NLR) on treatment delivery, toxicity, and survival.

**Methods:**

We retrospectively analyzed patients with locally advanced or metastatic pancreatic adenocarcinoma treated with first-line GnP at a tertiary center. Composite frailty was defined as an mFI ≥2 and a PNI <45. The primary endpoint was reduced relative dose intensity (RDI <75%) during the first 8 weeks. Secondary endpoints included time-to-discontinuation (TTD), overall survival (OS), severe toxicities, and the prognostic value of NLR cutoffs (≥3, ≥5).

**Results:**

Among 114 patients, 34 (29.8%) had composite frailty. Composite frailty was associated with reduced RDI <75% (odds ratio [OR] 2.65, 95% confidence interval [CI] 1.02–7.16, p=0.049), but not with severe adverse events, TTD, or OS. Higher NLR was associated with shorter TTD and worse OS. Secondary analyses showed that in frail patients, NLR ≥5 (but not ≥3) predicted inferior OS (hazard ratio [HR] 3.11, 95% CI 1.34–7.21, p=0.008). In non-frail patients, both NLR ≥3 and ≥5 were significantly associated with poor OS.

**Conclusions:**

To our knowledge, this study is among the first to collectively evaluate composite frailty and NLR in pancreatic cancer patients treated with GnP. Frailty was mainly associated with chemotherapy delivery, whereas NLR provided stronger prognostic information for survival. These complementary markers may support treatment optimization and personalized care for vulnerable patients.

## 1 Introduction

Pancreatic cancer is one of the most aggressive gastrointestinal malignancies (1, 2). Most patients present with locally advanced or metastatic disease, and systemic chemotherapy is the mainstay of treatment (3, 4). Current guidelines recommend FOLFIRINOX or gemcitabine plus nab-paclitaxel (GnP) as first-line regimens (5, 6). In practice, GnP is often selected for patients with a less favorable performance status and administered with modified schedules or dose reductions to improve tolerability (7–9). Older and vulnerable patients, who account for much of the pancreatic cancer population, remain underrepresented in clinical trials (10, 11). Consequently, the optimal regimens and dosing strategies for this group remain unclear.

Therefore, frailty assessment has been a focus of research in gastrointestinal oncology (12–15). However, available tools are diverse and inconsistent, and many emphasize only comorbidities or functional decline (12, 14, 16–18). Their application has been limited by a lack of standardization, modest clinical feasibility, and a shortage of geriatric specialists. In gastrointestinal cancers, simple markers such as the modified frailty index (mFI-5) and the prognostic nutritional index (PNI) have been validated as predictors of treatment outcomes (19–23). More recently, systemic inflammation, commonly measured using the neutrophil-to-lymphocyte ratio (NLR), has been recognized as another dimension of vulnerability, reflecting tumor biology and the host immune response (24, 25). In pancreatic cancer, low PNI and elevated NLR have been associated with poor survival and high treatment-related morbidity (20, 26, 27).

Despite these findings, the roles of frailty and biomarkers in patients receiving GnP have not yet been fully established. Whether frailty primarily affects chemotherapy delivery, such as the relative dose intensity (RDI), or directly influences outcomes, including time-to-discontinuation (TTD) and survival, remains unclear. To address this gap, we evaluated a composite frailty measure that integrates both functional (mFI) and nutritional (PNI) domains. We also examined the prognostic impact of systemic inflammation using NLR in patients with locally advanced or metastatic pancreatic cancer treated with GnP.

## 2 Methods

### 2.1 Study design

This retrospective, single-center study was conducted at Korea University Anam Hospital, a tertiary referral center in Seoul, Korea. We reviewed consecutive patients with biopsy-confirmed locally advanced or metastatic pancreatic adenocarcinoma who received GnP as first-line palliative chemotherapy between January 2016 and January 2025. The decision to initiate systemic chemotherapy was made through multidisciplinary team discussions involving surgeons, radiologists, and medical oncologists.

### 2.2 Patients and data collection

Eligible patients were adults (≥19 years). Exclusion criteria included unavailability of essential baseline data required for frailty assessment and discontinuation of therapy before completing the first cycle for non-medical reasons. Pregnant patients and those younger than 18 years were not eligible for the study.

Baseline variables included age, sex, Eastern Cooperative Oncology Group performance status (ECOG PS), metastatic status, prior biliary drainage, and initial dose reduction. Laboratory variables included serum albumin levels, absolute lymphocyte counts, and neutrophil counts. The PNI was calculated as 10 × albumin [g/dL] + 0.005 × lymphocyte count [/mm³], with values <45 considered low (28, 29). Lymphocyte counts were recorded in units of/mm³ (equivalent to/μL) for all patients. The neutrophil-to-lymphocyte ratio (NLR) was calculated by dividing the neutrophil count by the lymphocyte count.

Frailty was assessed using the mFI-5, which assigns one point each to diabetes, hypertension requiring medication, chronic obstructive pulmonary disease, congestive heart failure, and functional dependence in activities of daily living (22, 30). Patients were categorized as frail if their score was ≥2 (30, 31). For the main analysis, a composite frailty variable was defined as the coexistence of frailty using an mFI ≥2 and low PNI (<45), aiming to capture both functional decline and nutritional impairment as components of vulnerability. NLR was analyzed both as a continuous variable and by prespecified cutoffs (≥3 and ≥5), which were selected based on previous research demonstrating prognostic relevance in pancreatic and other gastrointestinal cancers (32, 33).

### 2.3 Treatment regimen

At our institution, the protocol-defined standard regimen consists of gemcitabine (1000 mg/m²) and nab-paclitaxel (125 mg/m²) administered on days 1, 8, and 15 of a 28-day cycle. For the purpose of the present study, the RDI was calculated against this uniform protocol-defined dose, which was identical for all patients. However, in clinical practice, dose reductions or delays can be applied at the discretion of the treating physician based on the patient’s performance status, comorbidities, or treatment-related toxicities. Treatment data included the actual weekly doses of gemcitabine and nab-paclitaxel administered during the first 8 weeks, from which the RDI was derived.

### 2.4 Outcomes

The primary outcome was the reduced RDI during the first 8 weeks (<75%), calculated relative to the protocol-defined standard dose (34, 35). Secondary outcomes included TTD, overall survival (OS), and treatment-related adverse events (AEs). OS was defined as the time from treatment initiation to death from any cause. TTD was defined as the time from treatment initiation to the permanent discontinuation of GnP, regardless of the cause (disease progression, toxicity, or patient decision). AEs were graded according to the Common Terminology Criteria for Adverse Events (version 5.0). Treatment-related hospitalizations were also recorded (36).

### 2.5 Statistical analysis

Baseline characteristics are summarized as mean ± standard deviation or median [interquartile range (IQR)] for continuous variables, and as counts (percentages) for categorical variables. Groups were defined by the composite frailty status (mFI ≥ 2 and PNI < 45). Between-group comparisons were performed using Student’s t-test or Wilcoxon rank-sum test for continuous variables and the chi-squared or Fisher’s exact test for categorical variables.

Early treatment delivery and toxicity were described using summary tables that included grade ≥3 adverse events within 8 weeks, RDI during the first 8 weeks (both continuous and <75% binary), and the 8-week treatment discontinuation indicator.

To evaluate independent associations, multivariable models were fitted with the composite frailty as the main exposure: logistic regression for reduced RDI (<75%) and for grade ≥3 adverse events, and Cox proportional hazards models for TTD and OS. All models were adjusted for age, sex, ECOG PS (≥2 vs. <2), metastatic status, prior biliary drainage, initial dose reduction at the initiation of treatment, and NLR (log-transformed to account for skewed distribution). The results are presented as odds ratios (ORs) or hazard ratios (HRs) with 95% confidence intervals (CIs).

Kaplan–Meier curves for TTD and OS were compared using the log-rank test, and the median survival with 95% CIs was reported. Adjusted effect estimates for the composite frailty across endpoints (RDI<75%, grade ≥3 AEs, TTD, OS) were summarized in a forest plot.

In secondary analyses, the prognostic impact of systemic inflammation was evaluated using established NLR cutoffs (≥3 and ≥5). Cox proportional hazards models for OS were fitted separately within the composite frail and non-frail subgroups, which is a statistical method consistent with previous studies.

For supplementary analyses, we performed joint stratification by composite frailty (mFI ≥2 and PNI <45) and NLR cutoffs (≥3 and ≥5). Patients were categorized into four groups (non-frail/low-NLR, non-frail/high-NLR, frail/low-NLR, and frail/high-NLR), and Kaplan–Meier survival curves with log-rank tests (false discovery rate-adjusted for pairwise comparisons) were generated. Median OS and 95% CIs were estimated for each subgroup.

All analyses were conducted using R version 4.4.3 (R Foundation for Statistical Computing, Vienna, Austria); two-sided p-values <0.05, were considered statistically significant.

### 2.6 Ethics

This study was approved by the Institutional Review Board of Korea University Anam Hospital (2025AN0462). The requirement for informed consent was waived owing to the retrospective study design, and all patients were treated in accordance with standard clinical practice.

## 3 Results

### 3.1 Patient characteristics by frailty–nutritional status

Baseline characteristics of the patients are shown in **Table 1**. Of 114 patients, 34 (29.8%) were classified as composite frail (mFI ≥2 & PNI <45) and 80 (70.2%) as composite non-frail. Frail patients were older (mean age 78.3 vs. 74.4 years, p=0.015) and more likely to have poor performance status (ECOG PS ≥2:20.6% vs. 2.50%, p<0.001). Initial dose reduction was more frequent in the frail group (58.8% vs. 25.0%, p<0.001). In addition, frail patients had lower albumin and lymphocyte counts and PNI and higher NLR values. Other baseline characteristics, including sex distribution, metastatic status, and prior biliary drainage, were comparable between the groups.

**Table 1:** Baseline characteristics by frailty–nutritional status.

Variable	Composite frail (mFI≥2 & PNI<45) N = 34	Composite non-frail N = 80	p-value
Age (years), mean ± SD	78.3 ± 7.1	74.4 ± 7.9	0.015
Sex (Female/Male), %			0.6
Female	18 (52.9%)	38 (47.5%)	
Male	16 (47.1%)	42 (52.5%)	
ECOG PS			<0.001
0	2 (5.9%)	27 (33.75%)	
1	25 (73.5%)	51 (63.75%)	
2	7 (20.6%)	2 (2.50%)	
Metastatic disease	33 (97.1%)	72 (90.0%)	0.3
Prior biliary drainage			0.9
No	28 (82.35%)	65 (81.25%)	
Yes	6 (18.65%)	15 (18.75%)	
Initial dose reduction			<0.001
No	14 (41.2%)	60 (75.0%)	
Yes	20 (58.8%)	20 (25.0%)	
Initial DR level			0.007
-40%	1 (2.9%)	1 (1.25%)	
-20%	17 (50.0%)	19 (23.75%)	
No dose reduction	16 (47.1%)	60 (75.0%)	
Albumin (g/dL)	3.20 (2.90, 3.40)	3.85 (3.35, 4.10)	<0.001
Lymphocyte (/mm³)	1,072 (873, 1,581)	1,738 (1,346, 2,150)	<0.001
Neutrophil (/mm³)	5,530 (4,500, 6,518)	4,838 (3,447, 6,905)	0.7
Prognostic Nutritional Index	39 (34, 41)	48 (41, 51)	<0.001
Neutrophil-to-lymphocyte ratio	4.5 (3.1, 7.0)	2.9 (1.8, 5.7)	0.012

ECOG PS, Eastern Cooperative Oncology Group performance status; SD, standard deviation; mFI, modified frailty index; PNI, prognostic nutritional index; DR, dose reduction.

Wilcoxon rank-sum test; Pearson’s chi-squared test; Fisher’s exact test.

### 3.2 Impact of composite frailty on RDI, toxicity, and early discontinuation

Patients with composite frailty (mFI ≥2 & PNI <45) showed lower early treatment intensity than did those without composite frailty (**Table 2**). The median 8-week RDI was 0.63 (IQR 0.17–0.95) in the composite frail group and 0.83 (0.50–1.00) in the non-frail group (p=0.015), and the proportion of patients with an RDI <75% was higher in those with composite frailty (64.7% vs. 36.25%; p=0.005). Rates of grade ≥3 toxicity within 8 weeks were numerically higher in the composite frail group (85.3% vs. 72.5%) but did not reach statistical significance (p=0.14). Early treatment discontinuation (≤56 days) occurred in 52.9% of patients with composite frailty vs. 41.25% of those without (p=0.30), and the median time-to-discontinuation was 53 days (IQR, 8–162) versus 71 days (IQR, 38–158), respectively (p=0.20).

**Table 2:** Early clinical outcomes within the first 8 weeks by composite frailty classification.

Variable	Composite frail (mFI≥2 & PNI<45) N = 34	Composite non-frail N = 80	p-value
Incidence of grade ≥3 treatment-related toxicity within 8 weeks			0.14
No	5 (14.7%)	22 (27.5%)	
Yes	29 (85.3%)	58 (72.5%)	
Relative dose intensity (RDI) during the first 8 weeks	0.63 (0.17, 0.95)	0.83 (0.50, 1.00)	0.015
Suboptimal dose intensity (<75%) during the first 8 weeks			0.005
No	12 (35.3%)	51 (63.75%)	
Yes	22 (64.7%)	29 (36.25%)	
Early treatment discontinuation within 8 weeks			0.3
No	16 (47.1%)	47 (58.75%)	
Yes	18 (52.9%)	33 (41.25%)	
Median time-to-discontinuation (days)	53 (8, 162)	71 (38, 158)	0.2

mFI, modified frailty index; PNI, prognostic nutritional index.

### 3.3 Survival outcomes by composite frailty

**Figure 1:**
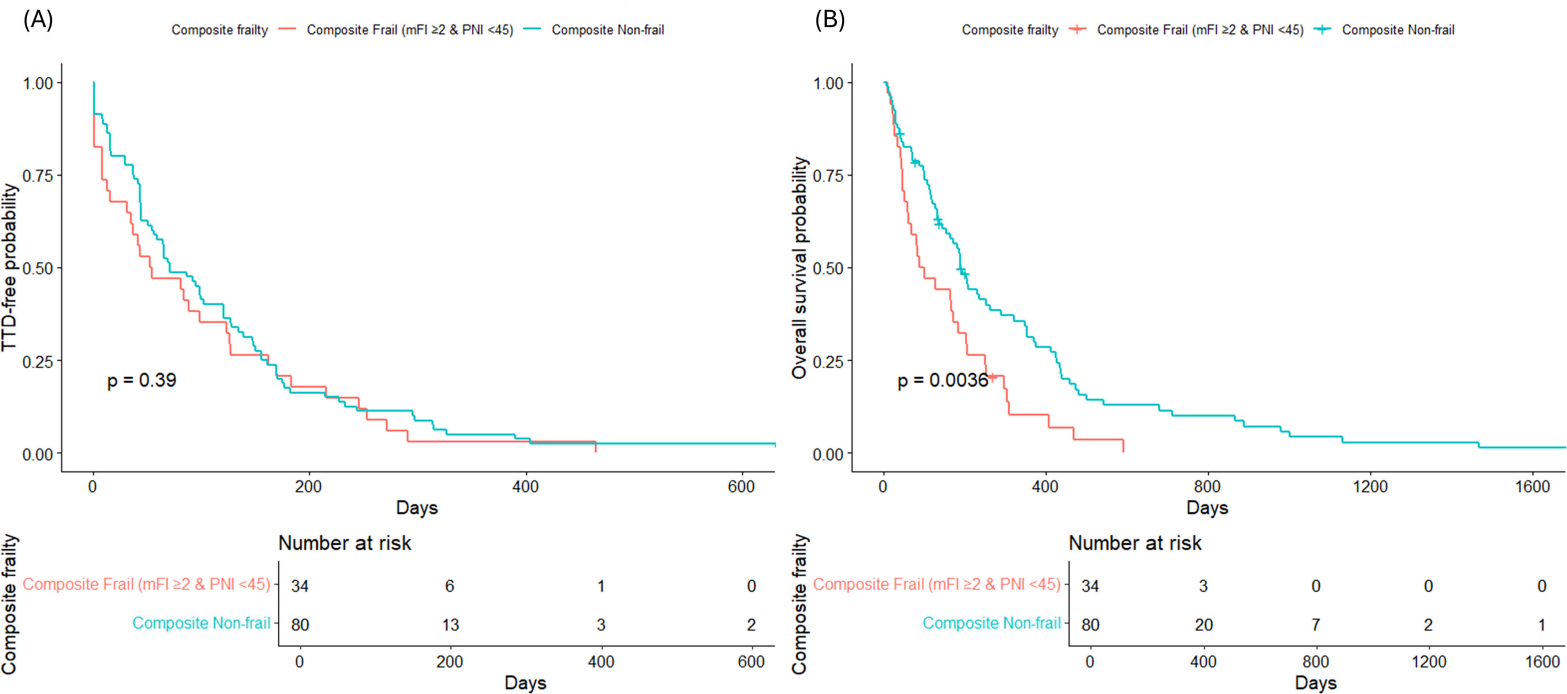
Kaplan–Meier curves for time-to-discontinuation and overall survival according to composite frailty status. Patients with composite frailty (mFI ≥2 & PNI <45) showed shorter OS compared with non-frail patients (p=0.0036, log-rank test). No significant difference in time to discontinuation was observed between the groups (p=0.39). The at-risk numbers are shown in each plot. TTD, time-to-discontinuation; mFI, modified frailty index; PNI, prognostic nutritional index; OS, overall survival.

Kaplan–Meier analysis revealed that composite frailty was associated with inferior OS but not with TTD (**Figure 1**). The median TTD was 53 days (95% CI, 35–126) in the composite frail group and 71 days (95% CI, 56–120) in the non-frail group (log-rank p=0.39). In contrast, median OS was significantly shorter in patients with composite frailty than that in those without (93.5 days, 95% CI 62–203 vs. 189 days, 95% CI 154–288; log-rank p=0.0036).

### 3.4 Multivariable analyses of composite frailty and outcomes

In multivariable analyses adjusting for clinical and laboratory covariates, composite frailty (mFI ≥2 and PNI <45) was significantly associated with reduced treatment intensity (RDI <75%) (**Table 3**). Patients with composite frailty had higher odds of receiving RDI <75% within the first 8 weeks (OR 2.65, 95% CI 1.02–7.16, p=0.049). In contrast, composite frailty was not associated with ≥grade 3 AEs (OR 1.48, 95% CI 0.44–5.59, p=0.50), time-to-discontinuation (HR 1.06, 95% CI 0.68–1.65, p=0.80), or OS (HR 1.46, 95% CI 0.92–2.32, p=0.11).

**Table 3:** Multivariable analysis of predictive factors for toxicity, treatment delivery, and survival.

Variable	≥G3 AE			RDI<75%			TTD			OS		
	OR	95% CI	p-value	OR	95% CI	p-value	HR	95% CI	p-value	HR	95% CI	p-value
Composite frail (mFI≥2 & PNI<45)	1.48	0.44, 5.59	0.5	2.65	1.02, 7.16	0.049	1.06	0.68, 1.65	0.8	1.46	0.92, 2.32	0.11
Age	1.05	0.99, 1.13	0.14	1.02	0.97, 1.08	0.5	0.99	0.96, 1.01	0.4	1	0.98, 1.03	0.8
Sex: Female	0.43	0.15, 1.14	0.1	0.57	0.24, 1.31	0.2	1.14	0.77, 1.69	0.5	0.98	0.65, 1.46	>0.9
ECOG PS ≥2	1.8	0.25, 37.1	0.6	2.9	0.58, 21.6	0.2	1.5	0.71, 3.14	0.3	1.8	0.85, 3.78	0.12
Metastatic disease	0.18	0.01, 1.43	0.2	0.67	0.14, 3.15	0.6	1.45	0.68, 3.07	0.3	1.31	0.60, 2.87	0.5
Starting dose reduction	1.01	0.33, 3.15	>0.9	0.91	0.35, 2.27	0.8	0.89	0.57, 1.39	0.6	1.05	0.68, 1.64	0.8
Prior biliary drainage	3.76	0.90, 26.3	0.11	2.12	0.75, 6.26	0.2	1.26	0.76, 2.09	0.4	1.18	0.71, 1.98	0.5
lnNLR	1.87	0.98, 3.83	0.069	1.37	0.80, 2.39	0.3	1.5	1.14, 1.97	0.003	1.85	1.36, 2.50	<0.001

AE, adverse event; ≥G3 AE, grade 3 or higher adverse events; mFI, modified frailty index; PNI, prognostic nutritional index; RDI, relative dose intensity; TTD, time to treatment discontinuation; OS, overall survival; OR, odds ratio; HR, hazard ratio; CI, confidence interval; ECOG PS, Eastern Cooperative Oncology Group performance status; lnNLR, log-transformed neutrophil-to-lymphocyte ratio.

Among covariates, higher log-transformed NLR was significantly associated with shorter TTD (HR 1.50, 95% CI 1.14–1.97, p=0.003) and worse OS (HR 1.85, 95% CI 1.36–2.50, p<0.001), whereas other clinical factors, including age, sex, ECOG PS, metastatic status, initial dose reduction, and prior biliary drainage, were not significantly associated with outcomes.

**Figure 2:**
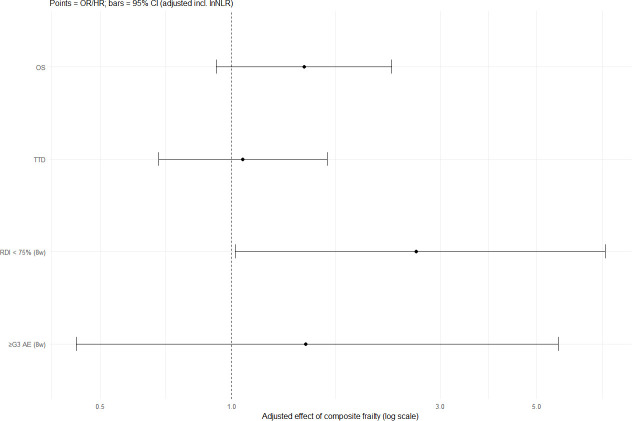
Forest plot of multivariate analyses for treatment toxicity, dose intensity, and survival. Forest plot summarizing multivariate logistic and Cox regression analyses for clinical outcomes, including incidence of grade ≥3 adverse events (AEs), suboptimal relative dose intensity (RDI <75%), time-to-treatment discontinuation (TTD), and overall survival (OS). Odds ratios (ORs) with 95% confidence intervals (CIs) are shown for binary outcomes (≥G3 AE, RDI <75%), and hazard ratios (HRs) with 95% CIs are shown for time-to-event outcomes (TTD, OS). The variables included composite frailty, age, sex, ECOG PS, metastatic disease, starting dose reduction, prior biliary drainage, and log-transformed neutrophil-to-lymphocyte ratio (lnNLR). ECOG PS, Eastern Cooperative Oncology Group performance status; NLR, neutrophil-to-lymphocyte ratio.

**Figure 2** shows a forest plot of the multivariable analyses. Composite frailty was significantly associated with reduced treatment intensity (RDI <75%). No significant associations were observed for grade ≥3 AEs, TTD, or OS.

### 3.5 Prognostic impact of NLR

Secondary, exploratory analyses were performed to evaluate the prognostic impact of the NLR according to frailty status (**Table 4**), given the limited sample size in these subgroups. Among patients with composite frailty, NLR ≥5 was associated with significantly worse OS (HR 3.11, 95% CI 1.34–7.21, p=0.008), whereas NLR ≥3 did not reach statistical significance (HR 2.10, 95% CI 0.81–5.42, p=0.126). In contrast, among non-frail patients, both NLR ≥3 (HR 2.04, 95% CI 1.25–3.33, p=0.004) and NLR ≥5 (HR 2.71, 95% CI 1.56–4.72, p<0.001) were significantly associated with inferior OS.

**Table 4:** Prognostic impact of NLR cutoffs on overall survival stratified by composite frailty.

Group	Cutoff	HR	95% CI	p-value
Composite frail group (mFI≥2 & PNI<45)	NLR ≥3	2.1	0.81–5.42	0.126
	NLR ≥5	3.11	1.34–7.21	0.008
Composite non-frail group	NLR ≥3	2.04	1.25–3.33	0.004
	NLR ≥5	2.71	1.56–4.72	<0.001

NLR, neutrophil-to-lymphocyte ratio; mFI, modified frailty index; PNI, prognostic nutritional index; HR, hazard ratio; CI, confidence interval.

In joint stratification by frailty and NLR, median OS was longest in non-frail patients with a low NLR (253 days, 95% CI 187–425) and shortest in frail patients with a high NLR (67 days, 95% CI 45–163). The intermediate groups (non-frail/high NLR and frail/low NLR) showed a median OS of 101 and 203 days, respectively. Pairwise log-rank tests confirmed a significant separation between the extreme groups (p<0.001, false discovery rate-adjusted), whereas the intermediate groups demonstrated modest separation (**Supplementary Figure 1**).

## 4 Discussion

Optimizing the delivery of palliative chemotherapy in frail patients with pancreatic cancer remains a major clinical challenge because treatment tolerance often limits the potential benefits of intensive regimens (1–4). Although effective regimens such as FOLFIRINOX and GnP are available, the optimal choice for first-line chemotherapy in clinical practice remains unclear (5, 6). This issue is particularly relevant in older or vulnerable patients, where efficacy must be weighed against tolerability. Therefore, frailty has been widely studied as a potential tool to guide treatment selection. However, the diversity of assessment methods has limited its clinical application (12, 18).

Our study extends previous research on this topic by evaluating frailty and systemic inflammation as prognostic factors of pancreatic cancer. Frailty, assessed using functional decline (mFI) and nutritional reserve (PNI), reflects patient-level vulnerability that influences chemotherapy delivery. In contrast, NLR captures systemic inflammatory activity and reduces lymphocyte-mediated immune competence, representing a biological pathway distinct from frailty. Previous studies have typically examined these domains in isolation, focusing on either frailty or nutritional indices, with limited integration of inflammatory markers (18, 22, 30). By combining both domains, our analysis highlights that frailty and NLR capture complementary dimensions of vulnerability rather than redundant measures, thereby providing a more comprehensive framework for risk stratification in the GnP setting.

Frail patients in our cohort received substantially lower treatment intensity, with a median RDI of 0.63 compared with 0.83 in the non-frail group (p=0.015), and a higher proportion of patients were classified as having suboptimal dose intensity <75% (65% vs. 36%, p=0.005). Despite the reduced dose delivery, the rates of severe toxicity and early discontinuation did not significantly differ. Survival analyses showed a similar pattern; frailty was associated with inferior OS in unadjusted Kaplan–Meier curves (median 93.5 vs. 189 days, log-rank p=0.0036), whereas TTD did not differ. However, in multivariable Cox regression, composite frailty was no longer independently predictive of survival but remained strongly linked to reduced RDI (OR 2.65, 95% CI 1.02–7.16, p=0.049). Collectively, these findings indicate that frailty exerts its impact mainly through treatment delivery rather than directly driving toxicity, discontinuation, or mortality. Notably, the number of patients at risk declined substantially at later follow-up time points; therefore, differences in long-term survival should be interpreted with caution.

The prognostic relevance of frailty in pancreatic cancer remains controversial (12, 14, 17, 18). Several studies have reported that frailty is closely linked to chemotherapy intolerance and poor survival in older adults with cancer, often outperforming chronological age or ECOG PS as prognostic markers for pancreatic cancer (12, 13, 37, 38). However, not all findings have been consistent, with some cohorts failing to demonstrate an independent association after adjustment for clinical and treatment-related factors (16–18). In our study, composite frailty was associated with reduced RDI but not with survival. One likely explanation is physician-driven dose tailoring conducted in clinical practice, in which clinicians proactively reduce or modify the starting doses in frail patients to preserve their tolerability. These findings raise the possibility that, when chemotherapy dosing is appropriately tailored based on frailty assessment in routine clinical practice, the direct adverse impact of frailty on survival may be attenuated. This observation supports the potential clinical value of frailty-guided personalized dose management. RDI was calculated against the protocol-defined standard regimen. Although our models were adjusted for baseline dose reduction, this factor may still serve as a marker of underlying frailty. The higher proactive dose reduction rate in frail patients suggests a clinical feedback loop that may attenuate the apparent prognostic effects of frailty on survival. Although RDI was calculated against a protocol-defined standard dose and our multivariable models adjusted for initial dose reduction, this variable likely reflects physician-driven assessment of patient frailty in routine clinical practice rather than an independent baseline characteristic. As such, initial dose reduction may act as an intermediate factor or proxy for perceived frailty, introducing residual confounding in estimating the association between composite frailty and chemotherapy delivery. This clinical feedback loop—whereby frailty influences starting dose decisions, which in turn affect measured RDI—may partially mediate the observed relationship between frailty and treatment delivery. Therefore, the independent effect of frailty on RDI should be interpreted with caution, and residual confounding cannot be fully excluded in this retrospective analysis. Another consideration is the heterogeneity of the frailty assessment methods across studies. While some reports have used comprehensive geriatric assessments or deficit accumulation indices, we employed a more pragmatic approach by combining the mFI with PNI, an approach which may be more feasible in routine oncology practice. In addition, methodological variability exists; prior cohorts differed in design and analytical endpoints, with some studies focusing primarily on quality of life outcomes rather than treatment delivery or survival (34).

Similar patterns have been reported for other gastrointestinal malignancies. Several studies in gastrointestinal malignancies have similarly reported that frailty indices are more closely associated with chemotherapy dose modification or treatment feasibility than with survival outcomes, particularly in real-world settings where proactive dose adjustment is commonly applied. In colorectal cancer, moderate dose reductions are often applied in older adult or frail patients receiving FOLFOX or FOLFIRI to improve tolerance (39–41). Several analyses have shown that such reductions do not significantly compromise OS, with no clear difference between full and reduced doses (39–42). In gastric cancer, frailty has been associated with poor long-term postoperative survival (15, 43). However, its impact on the chemotherapy setting appears less consistent, likely because clinicians proactively tailor doses (44). Further prospective multicenter studies with standardized frailty measures are required to clarify the prognostic role of frailty in patients with pancreatic cancer.

Systemic inflammation has also emerged as a strong prognostic factor (24, 27). NLR is a widely studied marker of host inflammatory and immune status, and elevated NLR levels have consistently been associated with poor outcomes across multiple malignancies, including pancreatic cancer (24, 26, 27). Previous studies suggest that an elevated NLR reflects a pro-tumorigenic microenvironment characterized by relative lymphopenia and neutrophil-driven immunosuppression (25, 45). In our cohort, a higher NLR was independently associated with a shorter TTD and worse OS, underscoring its prognostic relevance in the setting of GnP chemotherapy. Secondary analyses further demonstrated that the adverse prognostic impact of NLR ≥5 was particularly pronounced among composite frail patients, whereas both NLR ≥3 and ≥5 were predictive in non-frail patients. Joint stratification by frailty and NLR showed that survival was longest in non-frail/low-NLR patients and shortest in composite frail/high-NLR patients. Intermediate groups were moderately separated, supporting the concept that frailty and NLR capture complementary but distinct prognostic domains. However, subgroup sample sizes were relatively small and the potential for unstable estimates or type II errors should be acknowledged. Therefore, these exploratory subgroup findings should be interpreted with caution and confirmed using larger cohorts.

Our findings have several clinical implications. From a practical standpoint, assessment of composite frailty may help clinicians anticipate reduced treatment tolerance and proactively plan supportive care, closer monitoring, and dose modification strategies during GnP therapy. In contrast, elevated NLR, regardless of frailty status, may identify patients with particularly poor prognosis, for whom alternative approaches such as enrollment in clinical trials or consideration of more intensive systemic strategies could be discussed. Together, these findings suggest that frailty and inflammatory markers may inform complementary aspects of personalized treatment planning in advanced pancreatic cancer. Consistent with this framework, in composite frail patients—characterized by both functional decline (mFI) and impaired nutritional reserve (PNI)—baseline vulnerability was already substantial, and only high levels of inflammation (NLR ≥5) appeared to add further survival disadvantage. In contrast, among non-frail patients, even modest elevations in NLR (≥3) were suggestive of worse outcomes, implying that subtle immune-inflammatory dysregulation may influence survival despite preserved baseline function and nutrition. Clinically, this pattern supports the view that composite frailty and NLR may serve as complementary but distinct prognostic markers; composite frailty reflects treatment feasibility related to functional and nutritional reserve, and NLR captures aspects of tumor biology and host immune response. Therefore, the prognostic threshold of NLR may need to be interpreted within the context of frailty status. This concept requires confirmation in larger studies.

From a clinical perspective, evaluating composite frailty can help clinicians anticipate which patients are most likely to require dose modifications or supportive care during GnP therapy. By contrast, NLR offers a readily available measure of systemic inflammation that adds prognostic insight beyond frailty status. Using both assessments in combination may support more individualized decision-making, enabling physicians to balance treatment feasibility with expected survival outcomes in patients with advanced pancreatic cancer.

This study had several limitations. First, this was a retrospective single-center analysis with a modest sample size, which may limit generalizability and carries risks of selection bias and unmeasured confounding factors despite multivariable adjustment. As with any retrospective study, the study design precludes full control of all potential confounders, including details of supportive care practices and information on subsequent lines of therapy, which could have influenced treatment delivery and survival outcomes. Second, frailty was operationalized using a composite of mFI and PNI, with NLR evaluated as an additional covariate. Although this pragmatic approach reflects functional, nutritional, and inflammatory dimensions, mFI-5 and PNI do not capture other important geriatric domains such as cognitive function, psychological status, or social support, which may also influence treatment tolerance and outcomes. Comprehensive geriatric assessments incorporating these domains were not available, and alternative frailty indices or cutoffs may have yielded different results. Third, treatment delivery and toxicity data were retrospectively extracted from medical records. This may have led to an incomplete capture of lower-grade AEs, dose delays, or supportive interventions, thereby underestimating the true burden of treatment-related AEs. Fourth, the sample size limited the statistical power, particularly in the subgroup analyses of NLR cutoffs within the frail cohort, raising the possibility of a type II error. In addition, as this study was conducted at a tertiary referral center, the patient cohort may have been enriched with more complex or advanced cases, which could further limit the generalizability of the findings to other clinical settings. Finally, the long inclusion period spanning nearly a decade (2016–2025) may have introduced temporal heterogeneity related to changes in supportive care practices, patient selection, and treatment strategies over time. Such temporal variation could not be fully accounted for in the present analysis and should be considered when interpreting the results. Prospective multicenter studies with standardized frailty assessments are warranted to validate these findings.

In conclusion, composite frailty defined by mFI and PNI was associated with reduced chemotherapy delivery but not with survival outcomes, whereas systemic inflammation assessed using the NLR demonstrated stronger prognostic power. These findings suggest that integrating frailty and inflammatory markers provides a multidimensional view of vulnerability and could guide tailored chemotherapeutic strategies for patients with pancreatic cancer.

## Data Availability

The datasets supporting the conclusions of this article are available from the corresponding author on reasonable request.

## Glossary

GnP: gemcitabine plus nab-paclitaxel
mFI: modified frailty index
PNI: prognostic nutritional index
NLR: neutrophil-to-lymphocyte ratio
RDI: relative dose intensity
TTD: time-to-discontinuation
AE: adverse event
OS: overall survival
ECOG PS: Eastern Cooperative Oncology Group performance status
