# Supplementary material for: Frailty, nutritional status, and inflammation as determinants of chemotherapy delivery and outcomes in pancreatic cancer patients receiving gemcitabine plus nab-paclitaxel
Source: Front Oncol. 2026 Mar 16;16:1730394. doi: 10.3389/fonc.2026.1730394 (PMC13033535; doi:10.3389/fonc.2026.1730394)
Supplement: Supplementary file 1 [file DataSheet1.docx]

Supplementary Material

## Supplementary Figures


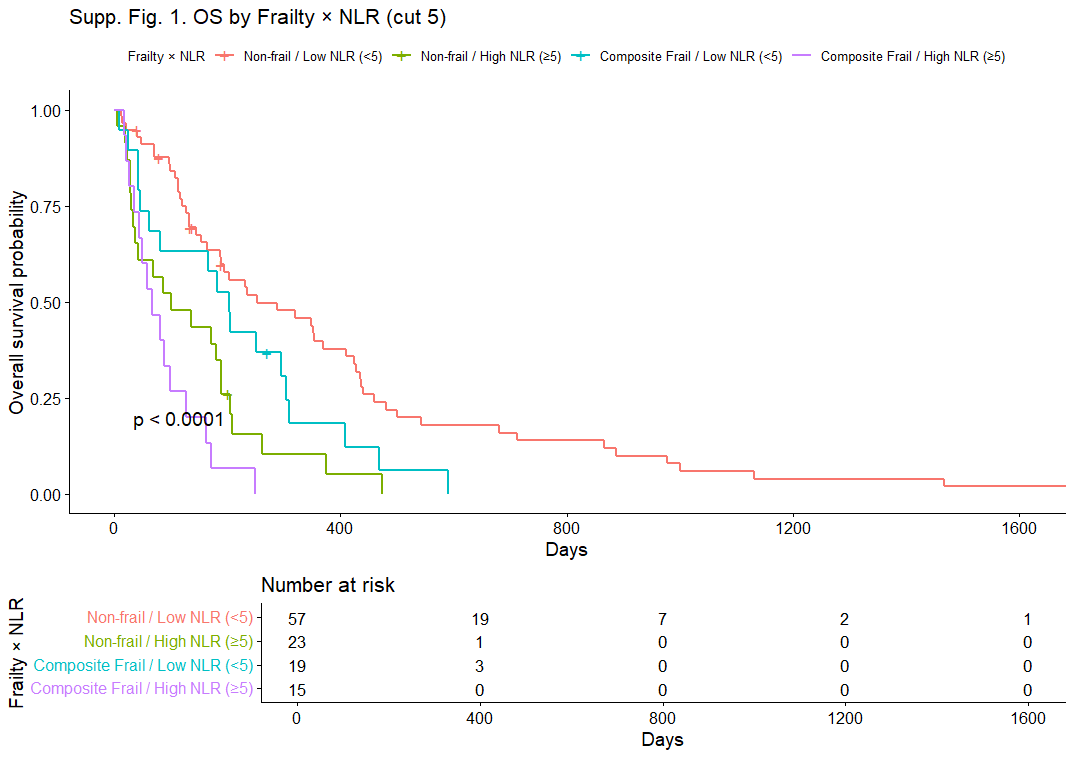


**(A)**


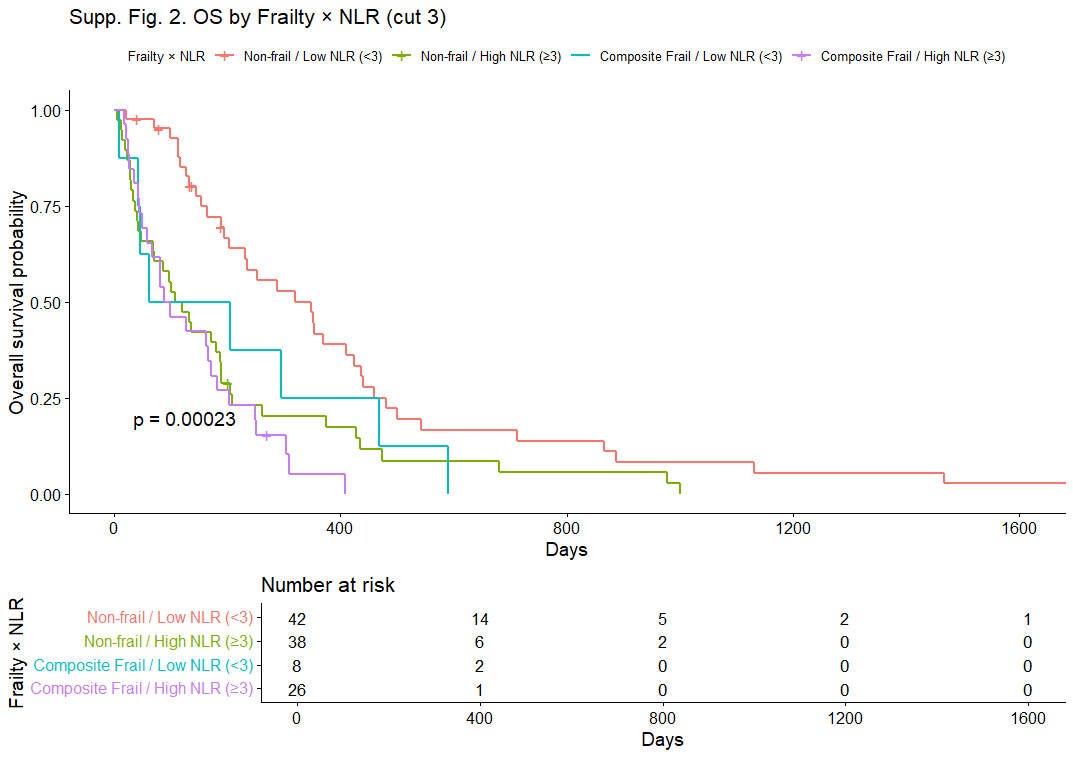


**(B)**

**Supplementary Figure 1.** Overall survival by frailty × NLR joint stratification

**(A)** Overall survival by frailty × NLR joint stratification (cutoff 5).

Kaplan–Meier curves are shown for the same four subgroups using an NLR cutoff of 5. Non-frail/low-NLR patients had the most favorable survival, whereas frail/high-NLR patients had the poorest outcomes. The two intermediate groups showed partial separation, consistent with frailty and NLR reflecting complementary but distinct prognostic domains. Pairwise log-rank tests with FDR adjustment were performed. Subgroup sample sizes were relatively small, particularly in the frail/high-NLR category, and findings should be interpreted with caution.

NLR, neutrophil-to-lymphocyte ratio; FDR, false discovery rate

**(B)** Overall survival by frailty × NLR joint stratification (cutoff 3).

Kaplan–Meier curves are shown for the four subgroups defined by composite frailty (mFI ≥2 and PNI <45) and NLR (cutoff 3). Median OS was longest in the non-frail/low-NLR group and shortest in the frail/high-NLR group. Differences between the intermediate groups were modest. Pairwise log-rank tests with FDR adjustment were used for comparison. Subgroup sizes were limited, particularly in the frail/low-NLR group, and results should be interpreted with caution.

NLR, neutrophil-to-lymphocyte ratio; mFI, modified frailty index; PNI, prognostic nutritional index; OS, overall survival
